# Supplementary material for: Validation of the Italian Version of the Behavioral Inhibition Questionnaire (BIQ) for Preschool Children
Source: Int J Environ Res Public Health. 2021 May 21;18(11):5522. doi: 10.3390/ijerph18115522 (PMC8196608; doi:10.3390/ijerph18115522)
Supplement: Supplementary file 1 [file ijerph-18-05522-s001.zip › ijerph-1193582-supplementary.pdf]

International Journal of
Environmental Research and Public Health

MDPI

Table S1. Fit Indexes for each Model for mother and father reports separately of Behavioral Inhibition Questionnaire (BIQ).

Model	GFI	NFI	PNFI	SRMR
**Mother BIQ**				
Model 1: 1 factor	0.93	0.91	0.84	0.09
Model 2: 3 correlated factors	0.94	0.92	0.85	0.08
Model 3: 6 correlated factors	0.97	0.96	0.86	0.06
Model 4: 6 first-order factors. 1 second-order factor	0.97	0.96	0.88	0.07
**Father BIQ**				
Model 1: 1 factor	0.92	0.89	0.82	0.09
Model 2: 3 correlated factors	0.93	0.90	0.83	0.09
Model 3: 6 correlated factors	0.96	0.94	0.84	0.07
Model 4: 6 first-order factors. 1 second-order factor	0.95	0.93	0.85	0.07

Note: GFI = Goodness of Fit Index; NFI = Normed Fit Index; PNFI= Parsimony Normed Fit Index; SRMR = Root Mean Square Error of Approximation.

Four models for parent reports were compared. The final model (fourth) is fully described in the main manuscript, while the results of the first three models are summarized below.

Model 1 (single factor): all the items significantly loaded the single factor, with 23 of the 30 BIQ items having a loading greater than 0.40 (items 4, 6, 13, 14, 17, 21 and 29 loaded below 0.40). However, as shown in Table 4, the one-factor solution did not provide good fit indices.

Model 2 (three correlated factors): all the BIQ items significantly loaded the three factors (Social novelty, Situational novelty, and Novel physical activities involving minor risk), and 27 of the 30 items showed a loading greater than 0.40 (items 4, 14 and 17 loaded below .40). Factor 1 (Social novelty) and factor 2 (Situational novelty) were strongly correlated (r = 0.77). In contrast, the correlation between factor 2 and factor 3 (Novel physical activities involving minor risk) and factor 1 and factor 3 were not significantly strong (r = 0.33 and r = 0.40, respectively). The comparison between the fit indices showed that the three-factor solution provided a better fit than the single factor model; however, the fit indices indicated a poor fit of the data (see NFI, GFI and RSMR indices in Table 4).

Model 3 (six correlated factors): the six correlated factor model (Peer situations, Physical challenges, Preschool/separation, Performing in front of others, Unfamiliar adults and Unfamiliar situations) provided a significantly better fit to the data compared to Model 2 (see fit indices, Table 4). The model showed a good fit to the data with a GFI higher than 0.95, NFI higher than 0.95, a PNFI higher than 0.50 and an SRMR lower than 0.08. All the BIQ items loaded on their designated factor with loading above 0.40 for 27 of the 30 items (only items 4, 14 and 17 loaded below 0.40). Latent variables were strongly positively correlated with each other (r greater than 0.30), except for the “Physical challenges” and “Unfamiliar adults” factors that have shown a r = 0.20 and “Physical challenges” and “Preschool/separation” that have shown a correlation index equal to 0.25. The overall CFA results provided support for a model in which BI responses clustered according to the six specific contexts.

However, it should be noted that item 4 and item 17 (“The child is cautious in activities that involve a physical challenge (e.g., climbing, jumping from a certain height,..)” and “The child is hesitant to explore new play equipment”, respectively) showed a low loading to the designated factor (0.39 and 0.22, respectively). Both items belonged to the same latent variable (Physical challenges) that also was the one less correlated to the other factors (correlation range was 0.20–0.48). Also, item 14 (“The child is independent”) showed a low loading to the designed factor (0.27) but belonged to the latent variable (Unfamiliar situations), which was highly correlated to the other factors (correlation range was 0.48–0.79).
